# Barium effect on germination, plant growth, and antioxidant enzymes in *Cucumis sativus* L. plants

**DOI:** 10.1002/fsn3.2177

**Published:** 2021-02-12

**Authors:** Noomene Sleimi, Rim Kouki, Maryem Hadj Ammar, Renata Ferreira, Rosa Pérez‐Clemente

**Affiliations:** ^1^ RME—Laboratory of Resources, Materials and Ecosystems Faculty of Sciences of Bizerte University of Carthage Bizerte Tunisia; ^2^ CERENA—Centro de Recursos Naturais e Ambiente Instituto Superior Técnico Universidade de Lisboa Lisboa Portugal; ^3^ Departamento de Ciencias Agrarias y del Medio Natural Universitat Jaume I Castellón Spain

**Keywords:** antioxidant enzymes, Ba accumulation, *Cucumis sativus* L., germination, plant growth

## Abstract

Barium (Ba) is a nonessential element that can cause several deleterious effects in most organisms. Elevated Ba concentrations can be toxic for plants and may affect growth and disturbances in homeostasis. This study aimed to evaluate the Ba stress, the plant‐tolerance limits, and the detoxification strategy adopted by *Cucumis sativus* L. The effect of Ba on seed's germination and vegetative development of this species was evaluated. For germination test, different Ba concentrations were used (0, 200, 500, 1,000, and 2,000 μM). Results showed that germination was stimulated with 500 and 2,000 µM of Ba. The toxicity effect on plant development was studied by treating the plants with increasing doses of Ba (100, 200, 300, and 500 μM) during 45 days. Shoot and root dry biomass production decreased significantly with elevated Ba concentrations, although water content enhanced in the roots. The concentration of Ba, 500 µM, induced high Ba accumulation in shoots and roots (9 times higher than in the control plants). Moreover, results showed that catalase, guaiacol peroxidase, and ascorbate peroxidase activities were stimulated in the different tissues of cucumber plants which highlight the occurring of an oxidative damage through Ba treatments and the involvement of the plant enzymatic antioxidant defense system.

## Graphical abstract

Barium treatments improve the germination percentage of cucumber seeds. Barium‐induced stress inhibits the growth of shoots and roots of cucumber plants. Water content increase in cucumber roots with elevated doses of barium. In cucumber plants barium accumulation occurs in both parts: shoots and roots. Barium treatments induce an increase in the activity of antioxidant enzymes (CAT, APX, and GPX).

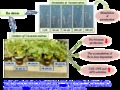

## 1 INTRODUCTION

Barium (Ba) is one of these contaminants, it is considered as the 14th most abundant element on Earth, and its concentration in soil ranges from 19 to 2,300 mg/kg, with average values of 265–835 mg/kg (Kabata‐Pendias, 2010). The toxicity of a Ba compound is significantly related to its solubility, and the more soluble the compound is, the more toxic it becomes (Lu et al., 2019). Its solubility in soil tends to increase with decreasing pH, and high cation exchange capacity (CEC) limits Ba mobility in the soil by adsorption (Madejón, 2013). Ba has been identified in over 80 minerals, but it occurs in significant quantities mainly in sparingly soluble forms such as barite (BaSO_4_) or witherite (BaCO_3_) (Boffito, 1991; DiBello et al., 1991). In fact, barium chloride is more toxic than barium carbonate owing to its high water solubility (Kravchenko et al., 2014). Although barite (component of fluids used in drilling of the oil and gas) has low solubility, it can still release amounts of Ba2+ in negatively charged soil colloids, posing a potential toxicity risk to plants and invertebrates (Lamb et al., 2013).

Several studies have indicated that plants showed different behavior in responses to abiotic stresses such as metal elements, salinity, and drought. For example, many halophytes are able to tolerate metal stress (Sleimi et al., 2014). However, *Sesuvium portulacastrum* L. growth decreased significantly at high salinity levels (600–1,000 mM) (Messeddi et al., 2001). Likewise, Ba exposure may cause multiple deleterious effects on plants. Raghu (2001) reported that around barium‐mining areas, high concentrations of Ba (500 µM) inhibited plant growth and potassium uptake in bush beans. Also, Ba treatments inhibited photosynthetic activity and plant growth in soybean plants (Suwa et al., 2008). The increase in Ba supply through nutrient solutions caused visible symptoms of Ba toxicity (like interveinal chlorosis and marginal necrotic spots in the leaf laminae) and sharply reduced the leaf area and dry‐mass yield of Tanzania Guinea grass (*Panicum maximum* Jacq.) (Monteiro et al., 2011).

Plants growing on Ba‐rich soils, around barite outcrops, or on mine spoils usually contain high Ba concentrations, although considerable differences between species have been reported. Barium concentrations in aboveground organs can be as high as or even higher than root Ba concentrations (Llugany et al., 2000). According to Raghu (2001), some plant species have adapted to high concentrations of TME and are able to survive in adversely impacted barite environments. Once accumulated in plant cells in rates above the threshold, TME, including Ba, cause the formation of reactive oxygen species (ROS). Indeed, ROS activate serious degradation of lipids, proteins, nucleic acid, and cellular antioxidants. As a response to oxidative damage, plants develop a natural antioxidant defense mechanism to counterbalance the ROS generated resulting from oxidative reactions, consisting at the production of enzymatic and nonenzymatic antioxidants (Ali et al., 2019). In order to protect cellular and sub‐cellular system from the cytotoxic effects of active oxygen radicals, antioxidant enzymes such as superoxide dismutase (SOD), catalase (CAT), guaiacol peroxidase (GPX), and ascorbate peroxidase (APX) are effectively involved (Siddiqi & Husen, 2017).

Although there is a lack of studies on Ba, absorption, and translocation over time, in plants with hyperaccumulatory potentials, the potential toxicity on plants grown in soils containing Ba still needs to be further investigated. In this context, the aim of this work was to assess the impact of the Ba‐induced stress and tolerance limits of *Cucumis sativus* plants. The study was designed to investigate the effect of Ba on germination, growth, and the involvement of the antioxidant enzyme activities such CAT, GPX, and APX in plant responses to BA stress.

## 2 MATERIAL AND METHODS

### 2.1 Plant material and culture

Prior the germination tests, the seeds of *Cucumis sativus* were soaked for 2 hr in distilled water, in order to ensure the lift of dormancy. Germination was performed in petri dishes with a double layer of filter paper fully moistened up with the test solutions made at different Ba doses: 0, 200, 500, 1,000, and 2,000 μM. The experiment was conducted in a growth chamber at 25°C during a period of 12 days, with aperiodic watering by treatment solutions to maintain the seed imbibition. The germination was followed after 24 hr of sowing with a daily count of germinated seeds (every 2 hr).

Plants were grown in a greenhouse of the Faculty of Sciences of Bizerte under natural photoperiod, relative humidity varied between 60% and 90%, and the temperature fluctuated between 12 and 25°C and regularly irrigated with Hewitt (1966) nutritive solution (3 times a week). After 30 days, plants were divided into 5 groups treated with different doses of Ba (0, 100, 200, 300, and 500 µM) added to the nutrient solution for 45 days.

In the harvest day, plants of each treatment were randomly divided into two groups, and as a first step, they were separated into roots and shoots and then washed with cold distilled water. Roots were dipped in a cold solution of CaCl_2_ (Stolt et al., 2003) to eliminate the adsorbed trace elements. For the first group, root and shoot fresh weights were immediately measured. The fresh samples were oven dried at 60°C for 10 days to measure the dry weights. For the second group, for each treatment, fresh plant material was divided into young leaves (collected below the stem apex), old leaves (harvested from the first internode), stems, and roots. The different plant tissues were crashed and frozen in liquid nitrogen and kept at −80°C for further analysis.

The determination of the fresh weight (FW) and the dry weight (DW) was carried out before and after drying as well as the water content (WC) that was determined as in Equation (1):

(1) $$\text{WC}=\left(\text{FW}‐\text{DW}\right)/{\text{DW and expressed in H}}_{2}{\text{O ml g}}^{‐1}\;\text{DW}$$

### 2.2 Germination parameters

The germination percentage (GP) was calculated by relating the number of seeds germinated to the total number of tested seeds (Ashraf & Abij‐Shakra, 1978) (Equation 2).

(2) GP=the number of seeds germinated/total number of seeds×100

Germination capacity (GCp) is the percentage of seeds that germinated during the germination process (Labouriau, 1983) and it was tested using the following equation (Equation 3):

(3) GCp=ni/N

with ni: the cumulative number of seeds germinated at each observation point. *N*: the total number of seeds that is set to germinate.

T_50_ is the time at which 50% of the germination is reached, and it is expressed as in Equation (4) (Salehzade et al., 2009):

(4) $$T_{50}=\text{ti}+\left(\frac{\left(N/2‐\text{ni}\right)\left(\text{tj}‐\text{ti}\right)}{\text{nj}‐\text{ni}}\right)$$

with *N*: the final number of seeds sprouted. ni_50_, nj_50_: the number of accumulated seeds corresponding to the time when ni < *N*/2 < nj. ti, tj: the time corresponding to ni and nj.

The germination velocity coefficient (GVC) is the reciprocal of the mean germination time (Equation 5) (Ranal & Garcia de Santana, 2006):

(5) $$\text{GVC}=\left(\frac{100\left(n1+n2+\cdots \text{nx}\right)}{n1t1+n2t2+\cdots \text{nxtx}}\right)$$

with nx: the number of seeds sprouted for an observation x. tx: the day corresponding to the germination of the seeds.

The germination index (GI) was calculated as described in the Association of Official Seed Analysts (AOSA, 1991) according to Equation (6):

(6) $$\text{GI}=\left(\frac{\text{nb of sprouted seeds}}{\text{the first day of counting}}+\ldots +\frac{\text{nb of sprouted seeds}}{\text{the last day of counting}}\right)$$

### 2.3 Measure of Ba accumulation

The mineralization was conducted during 2 hr at 110°C where the dry plant material was digested by mixture of acids (HNO_3_/H_2_SO_4_/HClO_4_; at the rate 10:1:0.5; v/v/v) (Sghaier et al., 2019). The obtained extracts were diluted by the nitric acid 0.5% and finally filtered to measure Ba content in plant tissues by atomic absorption spectrometry (Perkin Elmer PinAAcle 900T, USA).

### 2.4 Enzymatic assays

Enzymes extraction was carried out as follows: 400 mg of fresh plant material was grinded in 2 ml of extraction buffer (50 mM KH_2_PO_4_/K_2_HPO_4_ at pH 7.0, 5 mM Na ascorbate, and 0.2 mM EDTA). Subsequently, the homogenate was filtered through four layers of miracloth and centrifuged at 4830 *g* for 15 min at 4°C. The obtained supernatant was used to determine the activity of the antioxidant enzymes (CAT, APX, and APX).

The CAT activity was assayed at 240 nm by following the consumption of H_2_O_2_ by measuring the decrease in the optical density of a reaction mixture containing 50 μl of the protein extract, 50 mM H_2_O_2,_ and 25 mM potassium phosphate buffer (pH 7) as described in the protocol of Aebi (1984).

The enzymatic assay of GPX activity was performed according to the Fielding and Hall (1978). Briefly, the polymerization of guaiacol was followed measuring the increase in absorbance at 470 nm of the reaction mixture contained 10 µl of the protein extract, 30 mM H_2_O_2_, 25 mM phosphate buffer (pH 7), and 9 mM guaiacol.

The APX activity determination was carried out according to the Nakano and Asada (1981). The reaction is followed by measuring ascorbate consumption at 290 nm in a reaction mixture containing 40 μl of the protein extract, 2 mM H_2_O_2_, 25 mM potassium phosphate buffer (pH 7), 0.5 mM sodium ascorbate, and 0.1 mM EDTA. The activities are expressed as units of activity per milligram of protein in the crude extract (U g^−1^ DW).

### 2.5 Statistical analysis

All samples were analyzed for at least five replicates and mean values and standard deviation (±) are presented in vertical bars in the figures. The effects of TME on the variability of the studied parameters were evaluated using single‐factor analysis of variance (ANOVA1) by STATISTICA software to determine if a given factor has a significant effect. For the comparison of the means, the Tukey HDS test was used which gives the significant differences of these data at *p* < .05.

## 3 RESULTS

### 3.1 Germination parameters

**FIGURE 1:**
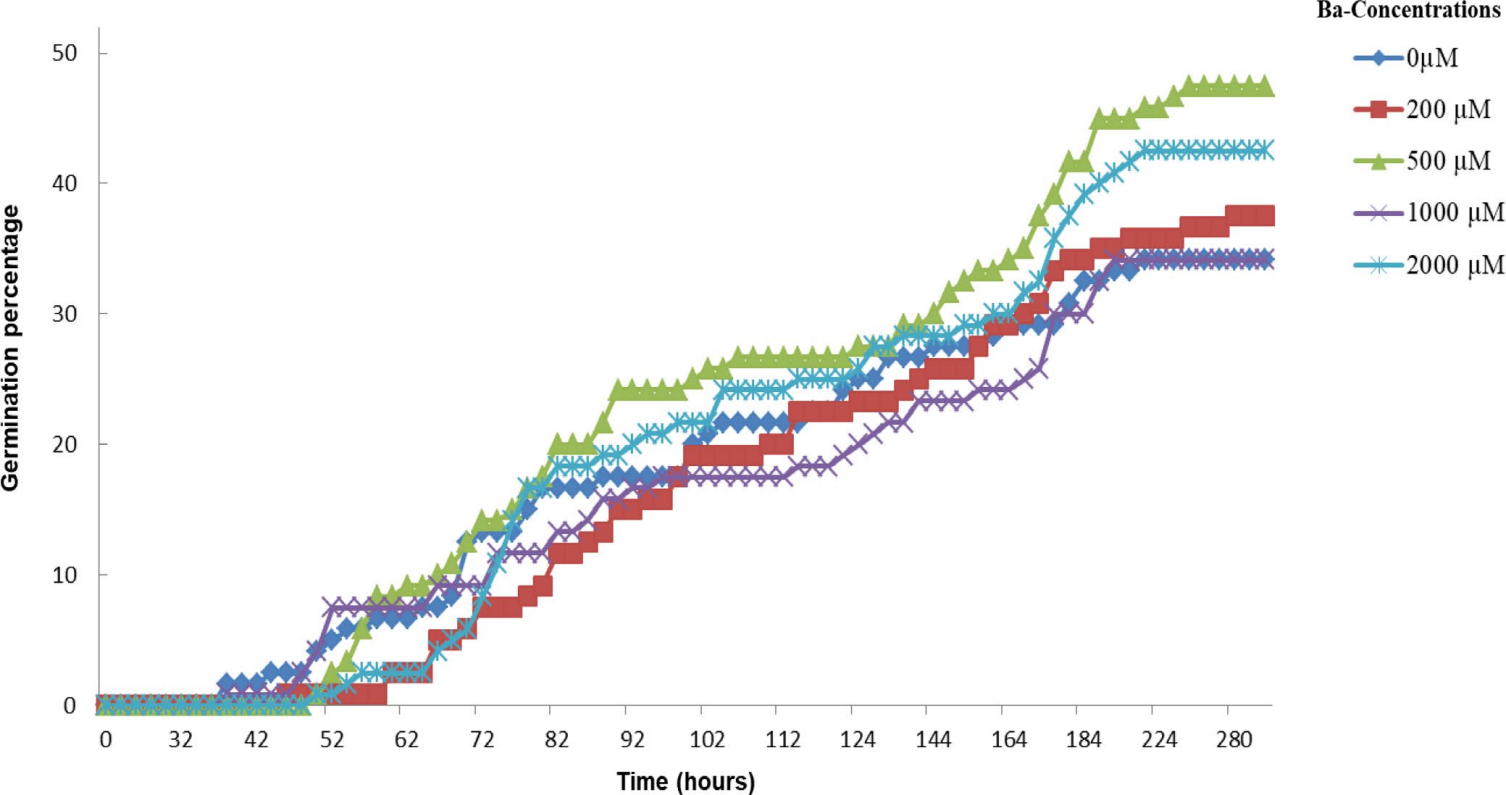
Effect of increasing doses of Ba (0, 200, 500, 1,000, and 2,000 μM) on the germination percentage (GP) of *Cucumis sativus* L. seeds

The germination of cucumber seeds was not negatively affected by Ba treatment. The best germination percentage was recorded in seeds treated with 500 µM (47.5%) and 2,000 µM (42.5%), while the lowest germination value was recorded in 1,000 µM (34.16%) similar to the control (34.2%) (Figure 1).

The data presented in Table 1 revealed that the longest T_50_ (109.71 hr) was recorded for 500 µM of Ba and the shortest T_50_ (94.75 hr) was verified for the control (0 μM of Ba), which means that this parameter is inversely correlated with the GP. The opposite results were recognized for GI and the GCp. These parameters are positively correlated with GP, where the highest GI and GCp values were recorded for 500 µM of Ba (7.48 and 0.23, respectively). On the other hand, our results show that the most important GVC (61.15) was reported in the control seeds. However, all the variations were not significant at *p* < .05 and consequently Ba had no effect on T_50,_ GCp and GVC.

**TABLE 1:** Variation of germination parameters (T50, GCp, GVC, GI) under the effect of Ba‐concentrations increasing (0, 200, 500, 1000 and 2000 µM). Different letters represent statistical differences at *p* ≤ 0.05

	0 µM	200 µM	500 µM	1,000 µM	2,000 µM
T_50_	94.8 ± 5.9^a^	100.8 ± 4.5^a^	109.7 ± 2.8^a^	99.0 ± 3.3^a^	109.3 ± 4.1^a^
CVG	61.2 ± 5.9^a^	56.9 ± 3.4^a^	56.6 ± 5.2^a^	52.8 ± 6.6^a^	50.2 ± 4.2^a^
GI	6.26 ± 0.50^ab^	5.64 ± 0.43^a^	7.48 ± 0.42^ab^	5.58 ± 0.33^a^	6.54 ± 0.50^ab^
GCp	0.18 ± 0.01^a^	0.17 ± 0.02^a^	0.23 ± 0.04^a^	0.17 ± 0.02^a^	0.20 ± 0.02^a^

### 3.2 Dry biomass production

**FIGURE 2:**
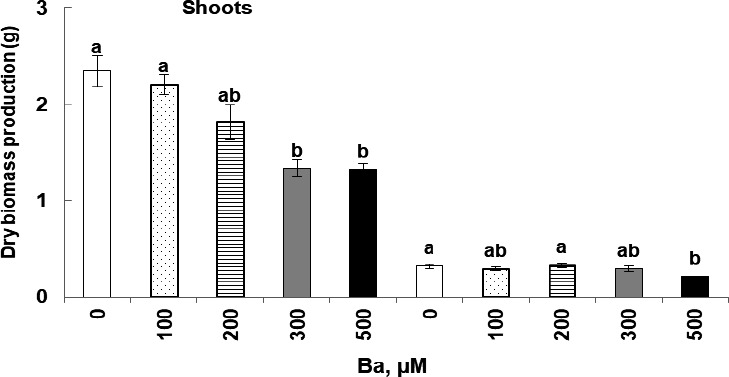
Variations of dry biomass production in shoots and roots of *Cucumis sativus* L. treated with 0, 100, 200, 300, and 500 μM of Ba. Data are mean values of 10 independent determinations ± *SE*. Different letters represent statistical differences at *p* ≤ .05

Our results show that in cucumber plants treated with increasing doses of barium the production of dry biomass was negatively affected (Figure 2). This decrease was noticed especially in the aerial parts even with the low doses. While 300 and 500 µM cause a significant decrease with reductions of 43.2% and 43.6%, respectively, compared with the control, similarly, the dry biomass in root was also negatively and significantly affected with 500 µM, with a reduction of 32.3% (*p* < .05).

### 3.3 Water content

**FIGURE 3:**
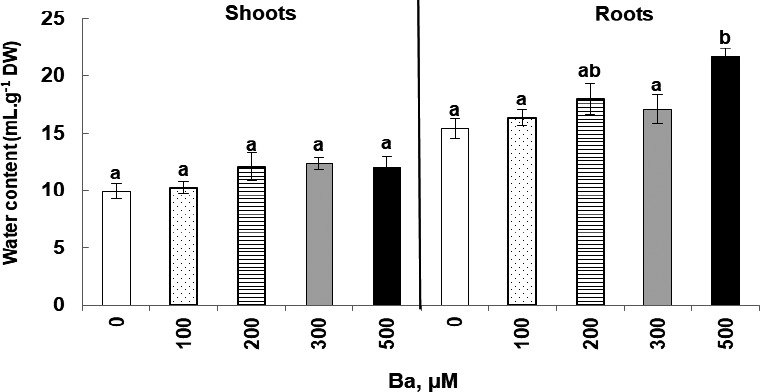
Variation of water content in shoots and roots of *Cucumis sativus* L. plants treated with 0, 100, 200, 300, and 500 µM of Ba. Data are mean values of 10 independent determinations ± *SE*. Different letters represent statistical differences at *p* ≤ .05

The variation of water content in shoots and roots of *Cucumis sativus* plants treated with Ba shows a slight improvement in the water status in shoots (Figure 3), especially with 200, 300, and 500 µM. The same trend was observed in the roots with a 1.5‐fold increase in the plants treated with 500 μM of Ba (21.7 against 15.4 ml g^−1^ DW in the control plants).

### 3.4 Barium content

**FIGURE 4:**
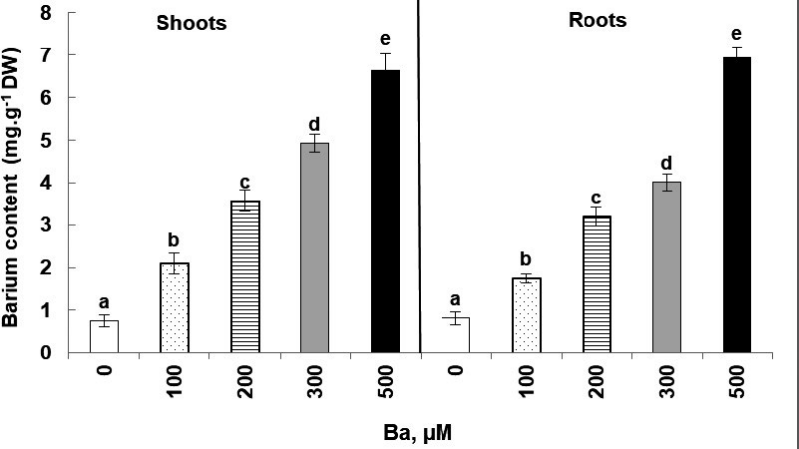
Variation of Ba contents in roots and shoots of *Cucumis sativus* L. plants treated with 0, 100, 200, 300, and 500 µM of Ba. Data are mean values of 10 independent determinations ± *SE*. Different letters represent statistical differences at *p* ≤ .05

As it is shown in Figure 4, the accumulation of barium in cucumber plant tissues was a dose dependent. Indeed, the increase in Ba content in tissues is proportional to the increase in Ba concentrations in the irrigation solution. It was also noticed that the accumulation took place in both parts, roots and shoots, and that both parts were able to retain the Ba with equal proportions.

In the aerial parts, the contents vary significantly (*p* < .05) from 0.74 mg g^−1^ DW for the control to 6.62 mg g^−1^ DW for plants treated with 500 μM of Ba. Similarly in roots, the contents vary significantly (*p* < .05) from 0.81 mg g^−1^ DW for the control to 6.93 mg g^−1^ for 500 µM of Ba. Results show an accumulation 9 times higher than the control in both plant tissues.

### 3.5 Antioxidant enzymatic activities

**FIGURE 5:**
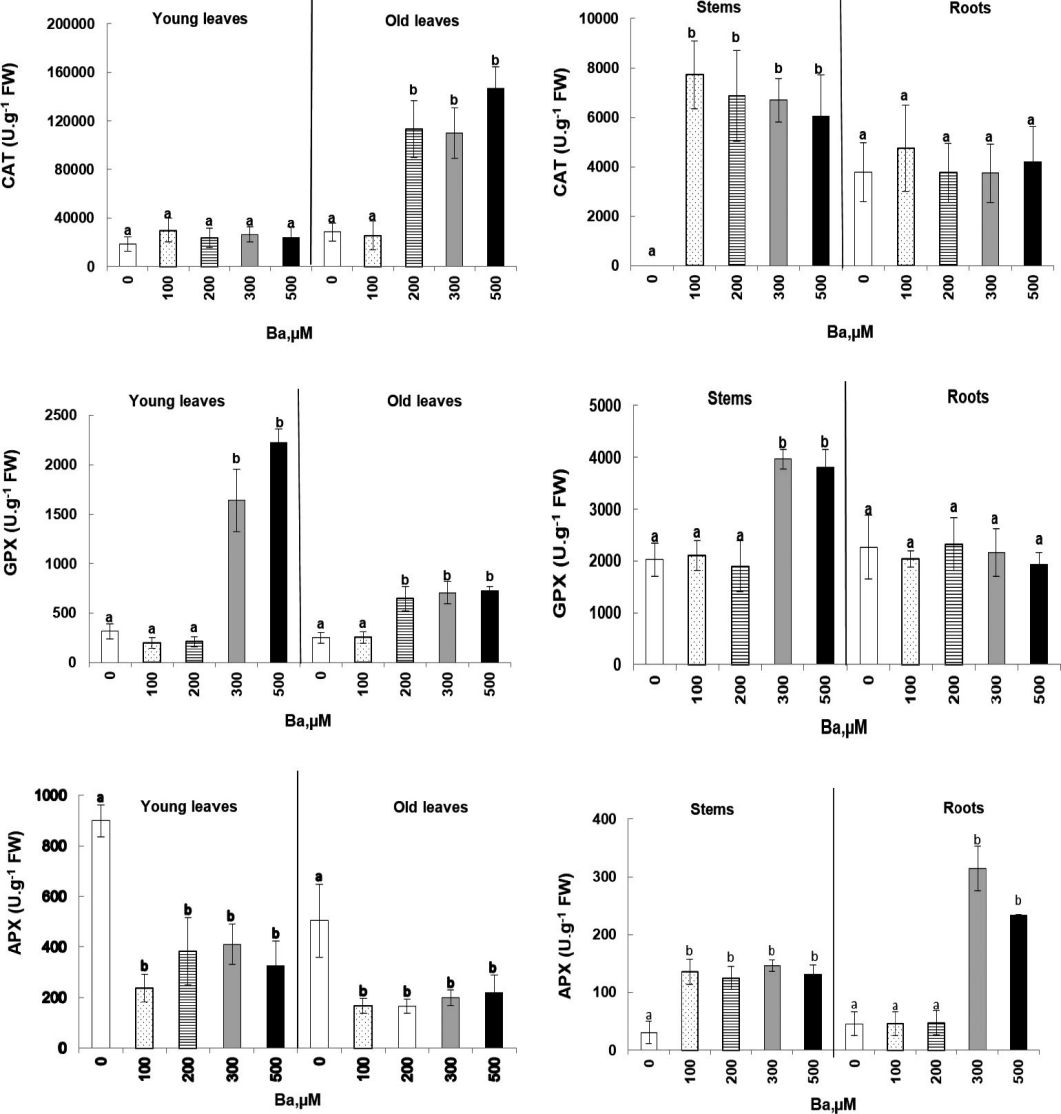
Variations of catalase (CAT), guaiacol peroxidase (GPX), and ascorbate peroxidase (APX) activities in young and old leaves, stems, and roots of *Cucumis sativus* L. plants treated with 0, 100, 200, 300, and 500 µM of Ba. Data are mean values of 10 independent determinations ± *SE*. Different letters represent statistical differences at *p* ≤ .05

In *Cucumis sativus*, the antioxidant enzymes undergo significant variations at *p* < .05 under the effect of treatment with the increasing doses of Ba (Figure 5). According to our results, CAT activity was stimulated in the aged leaves under the 200, 300, and 500 μM Ba treatment, exhibiting increases of 4.0‐, 3.9‐, and 5.2‐fold, respectively, compared with the control. The CAT activity also increased in the stems in all Ba treatments, although this increase is less significant than those obtained in the aged leaves. On the contrary, in young leaves and roots, no significant variation was reported under the Ba‐induced stress (Figure 5).

An increase in GPX activity was recorded after 3 min, especially with high doses of Ba. When we Compared to control, the young leaves showed an increase of 5.2‐ and 7.0‐fold with 300 and 500 µM, respectively. This increase was lower in the old leaves but still significant at *p* < .05. Similarly in the stems of plants treated with 300 and 500 µM of Ba, the GPX activity was significantly (*p* < .05) stimulated after 3 min, while no variation was noted for the roots (Figure 5).

The APX activity assay showed that the Ba treatment of cucumber plants induced significant inhibition in the young (55%–74%) and old leaves (57%–68%), regardless the dose used. On the contrary, APX activity was significantly stimulated in the stems (4.8‐ and 4.3‐fold increase) and roots (6.9‐ and 5.2‐fold increase) in plants treated with 300 and 500 µM of Ba.

## 4 DISCUSSION

### 4.1 Germination

Inappropriate conditions may compromise the ability of seeds to sprout. It has been proven that thermal stress and drought stress affected germination parameters in four chickpea varieties (Sleimi et al., 2013). In fact, germination and seedling development are the most sensitive physiological stages in plants, especially under metallic stress, since the defense processes are affected, being often regarded as an important index to evaluate plant tolerance to heavy metals (Talebi et al., 2014).

In this study, the assessment of the germination of *Cucumis sativus* seeds treated with increasing doses of Ba showed that the germination percentage was improved especially with 500 and 2,000 µM. This stimulation was also observed in *Cucurbita pepo* seeds treated with different concentrations of copper which shows an increase in the percentage of germination by 40% at 1,000 µM of Cu (Bankaji et al., 2017). Mahdieh et al. (2013) also had signaled that seed germination was stimulated in *Triticum aestivum* L. at low concentrations of arsenic comprised between 0 and 2.5 mg/L. Similarly, in *Vigna radiata* (L.) Wilczek and *Glycine max* (L.) Merr., 1 mg/kg arsenic addition stimulated seed germination and increased about 12% of the germination weight (Wan et al., 2013). Actually, this ability to tolerate the stress induced by metals in some plant species could be explained by the role played by the seed coat, which is a barrier between the embryo and the surrounding environment (Carlson et al., 1991). Despite the protecting role played by seed coat against the harmful effects of heavy metals, most seeds and seedlings show a decline in germination and vigor in response to heavy metal stress (Adrees et al., 2015). For example, Cd and Zn induced a decrease in the seed germination in cucumber (Wang et al., 2014) and 1 mM of Cd inhibited germination in *Picea omorika* (Prodanovic et al., 2016).

### 4.2 Growth

Barium is considered to be a nonessential element for organisms and is harmful to animals and plants (Lamb et al., 2013). In fact, it has been identified as a toxic element to most plants (Monteiro et al., 2011). Critical toxic concentrations of Ba in the substrate may largely vary with the Ba availability.

This study revealed that Ba negatively affected cucumber plant growth. Ba induced an inhibition of the dry biomass production along with the increase of Ba concentrations in the treatment solution. This repressor effect is worsened especially with the high doses (300 and 500 µM, Ba) in the aerial parts and in the roots.

Actually, few studies have focused on the toxicity induced by Ba in plants, and most of the studies have reported the inhibitory effect of this element on growth. In Tanzania Guinea grass*,* Ba caused a retarded growth besides of visible symptoms of toxicity such as interveinal chlorosis and marginal necrotic spots in the leaf laminae (Monteiro et al., 2011). Likewise, it caused a reduction in biomass production of bean (Llugany et al., 2000) and soybean (Suwa et al., 2008) grown in nutrient solution. This behavior was explained by the reduced of CO_2_ assimilation caused by limited photosynthetic activity in responses to abiotic stress (Caçador et al., 2016). In this case, Ba acts as an efficient K^+^‐channel blocker (Suwa et al., 2008). Ba also caused a reduction in protein concentration in soybean leaves after 30 and 45 days of exposure, which might also be a result of enhanced proteolysis (Melo et al., 2011).

Evidently, water is a requirement of living organisms but TME toxicity disturbs the water relationship of plants. Contrarily, our results reveled that water content was not reduced under the effect of Ba. In fact, water content increased specially in roots of plants treated with 500 µM. Other studies reveled that there was no significant effect of Ba treatment on water potential or relative water content (Suwa et al., 2008).

### 4.3 Barium content

Barium uptake by plants and its transport from roots to shoots may increase the exposure of humans and animals to Ba through vegetable or forage consumption. It has been proven that plants growing on Ba‐rich soils, around barite outcrops, or on mine spoils usually contain high Ba concentrations where the Ba concentrations in aboveground organs can be as high as or even higher than root Ba concentrations (Llugany et al., 2000).

In the same framework, our study showed that *Cucumis sativus* has a great susceptibility of Ba accumulation in different plant parts and with an equal distribution (the endogenous concentration of Ba increased with the increasing of the doses used in the irrigation solution). As a matter of fact, several plant species showed an adaptation against high concentrations of TME including Ba and were able to survive in adversely impacted barite environments. For example, *Indigofera cordifolia* can colonize, and accumulate Ba, at 3.5 mg g^−1^ DW (Raghu, 2001). *Cyperus papyrus* exported most of the Ba to the aerial part of the plant, especially at higher BaCl_2_ doses, while *Typha domingensis* accumulated preferentially in the roots (Ribeiro et al., 2018). In *Eleocharis acutangula*, the maximum accumulation of Ba occurred in the aerial parts of the plants at 105 days and in the roots at both 120 and 180 days (Ferreira et al., 2019). Actually, Ba was probably transported from the nutrient solution through the roots to the aboveground part. It can be hypothesized that free Ba was absorbed and readily transported in the upward movement of water in the xylem, in a way similar to that reported by Lombnaes and Singh (2003) for free manganese.

### 4.4 Antioxidant enzymes

One of the consequences of heavy metal or metalloids presence in plant cells is the formation of ROS. Indeed, plants can reduce their biomass production and may protect themselves from the negative effects through reactive oxygen species (ROS) (Sharma, 2013). The scavenging system to control ROS comprises of enzymatic and nonenzymatic components. Multiple enzymes including CAT, GPX, and enzymes of ascorbate‐glutathione (AsA‐GSH) cycle like APX interact in different subcellular components and respond when the plant is exposed to oxidative stress (Sharma et al., 2012).

The involvement of an antioxidant enzyme system, in response to Ba, has been proven in this assay. There was a variation in the activity of antioxidant enzymes in different parts of *Cucumis sativus* plants after a Ba treatment. CAT activity was stimulated in old leaves and stems. The same results were found in *Glycine max* L plants where CAT activity was expanded under Ba stress (Melo et al., 2011). Yang and Poovaiah (2002) suggest that the stimulation of this enzyme activity is closely linked to the increase in the intracellular concentration of hydrogen peroxide and Ca^2+^.

Similarly, high doses of Ba increased GPX activity in both young and old leaves and in the stems. Indeed, GPX activity is sensitive to the presence of TME within the cell, the latter are capable of modifying its activity, and it was proven that GPX activity was inhibited due to Cd and Cu treatments in *Suaeda fruticosa* Forsk. (Bankaji et al., 2015) and stimulated in *Atriplex halimus* L. with Cu (Bankaji et al., 2016).

Regarding the APX activity, the same behavior was observed in the stems and roots. On the other hand, in the young and aged leaves, there was an inhibition of the APX activity in stems and roots which can be explained by blocking functional groups, replacing essential metals with ETMs, changes in the structure or integrity of proteins, and disruption of the signal transduction of antioxidant enzymes (Alvarez & Lamb, 1997; Schützendübel & Polle, 2002; Stroinski & Kozlowska, 1997).

With this work, it was possible to conclude that Ba does not affect the germination of cucumber seeds. In fact, the germination percentage has even been improved with certain concentrations. On the other hand, in the plants, the dry biomass production was inhibited with high doses, especially in the aerial parts. It was also found that the cucumber exhibited a large capacity for accumulation of Ba in the roots and shoots. Also, the Ba‐induced stress has promoted the oxidative damage, which has been proven by the involvement of the antioxidant enzyme system namely with stimulation of CAT, GPX and APX activity.

## CONFLICT OF INTEREST

The authors have declared that no conflicts of interests exist.

## Data Availability

The data of this study are openly available in Food Science & Nutrition.
